# Incorporation of a unified protein abundance dataset into the *Saccharomyces* genome database

**DOI:** 10.1093/database/baaa008

**Published:** 2020-03-04

**Authors:** Robert S Nash, Shuai Weng, Kalpana Karra, Edith D Wong, Stacia R Engel, J Michael Cherry, SGD Project

**Affiliations:** Department of Genetics, Stanford University, 3165 Porter Drive, Palo Alto, CA 94304, USA

## Abstract

The identification and accurate quantitation of protein abundance has been a major objective of proteomics research. Abundance studies have the potential to provide users with data that can be used to gain a deeper understanding of protein function and regulation and can also help identify cellular pathways and modules that operate under various environmental stress conditions. One of the central missions of the *Saccharomyces* Genome Database (SGD; https://www.yeastgenome.org) is to work with researchers to identify and incorporate datasets of interest to the wider scientific community, thereby enabling hypothesis-driven research. A large number of studies have detailed efforts to generate proteome-wide abundance data, but deeper analyses of these data have been hampered by the inability to compare results between studies. Recently, a unified protein abundance dataset was generated through the evaluation of more than 20 abundance datasets, which were normalized and converted to common measurement units, in this case molecules per cell. We have incorporated these normalized protein abundance data and associated metadata into the SGD database, as well as the SGD YeastMine data warehouse, resulting in the addition of 56 487 values for untreated cells grown in either rich or defined media and 28 335 values for cells treated with environmental stressors. Abundance data for protein-coding genes are displayed in a sortable, filterable table on Protein pages, available through Locus Summary pages. A median abundance value was incorporated, and a median absolute deviation was calculated for each protein-coding gene and incorporated into SGD. These values are displayed in the Protein section of the Locus Summary page. The inclusion of these data has enhanced the quality and quantity of protein experimental information presented at SGD and provides opportunities for researchers to access and utilize the data to further their research.

## Introduction

The *Saccharomyces* Genome Database (SGD) collects, organizes and presents biological information about the genes and proteins of the budding yeast *Saccharomyces cerevisiae* (1–2). This information can be used to direct experimental research aimed at elucidating protein function and biological role in the context of the cell. Currently, Protein pages contain a descriptive overview of each predicted protein, experimental data such as protein abundance and half-life, structural domain information, primary amino acid sequence from a variety of strains with overlaid experimental post-translational modification (PTM) data, physico-chemical properties derived from the protein sequence, a list of external identifiers and links to other resources that may be of use to researchers. As a community resource, one of the core missions of SGD is to interact with users in a variety of ways to assess their needs and future research directions. One aspect of this interaction involves working with researchers to incorporate datasets of interest to the wider scientific community.

Although many genes are controlled at the transcriptional level, others are controlled translationally or post-translationally, and yet others, including rate-limiting regulators, are controlled at multiple levels. As a result, one goal of proteomics research is to reliably measure and quantitate protein content under standard growth conditions. Doing so provides researchers with context regarding abundance levels relative to other proteins in the proteome and provides baseline information that can then be extended to answer questions regarding the regulation of protein levels when cells are grown under stress conditions.

Recent advances in peptide labeling, sample preparation and both sensitivity and accuracy of mass spectrometry-based methods, coupled with advances in high-throughput imaging techniques, robotics and computational approaches to image analysis, have led to significant improvements in both the identification and quantification of proteins (3,4). These improvements have resulted in a proliferation of papers providing protein abundance datasets and provide an opportunity for the comprehensive analysis of protein abundance (5–25).

We became interested in updating our protein abundance data, with the goal of improving the quality and the quantity of experimental protein information available to our scientific community. As we surveyed the literature to collect protein abundance datasets, we concurrently learned that the laboratory of Grant Brown at the University of Toronto was collecting and evaluating protein abundance datasets with the goal of first normalizing the data presented in these studies and then converting it into the common units of molecules per cell (26). Since this dovetailed nicely with our desire to enhance experimental protein information presented on SGD Protein pages, we embarked on a collaborative effort to integrate this information and associated metadata into SGD. We also agreed that it would be advantageous to include median abundance values, and the Brown lab proposed calculating median absolute deviation (MAD). The median abundance provides a measure of the midpoint and makes it easier to compare the relative abundance of two or more proteins. The MAD provides a robust statistical measure of the variability within the abundance values. These values were added to Locus Summary pages for protein-coding genes and additionally integrated into the YeastMine data warehouse so that the abundance data and median values, even for large sets of genes, could be easily retrieved with templated queries (27).

**Figure 1:**
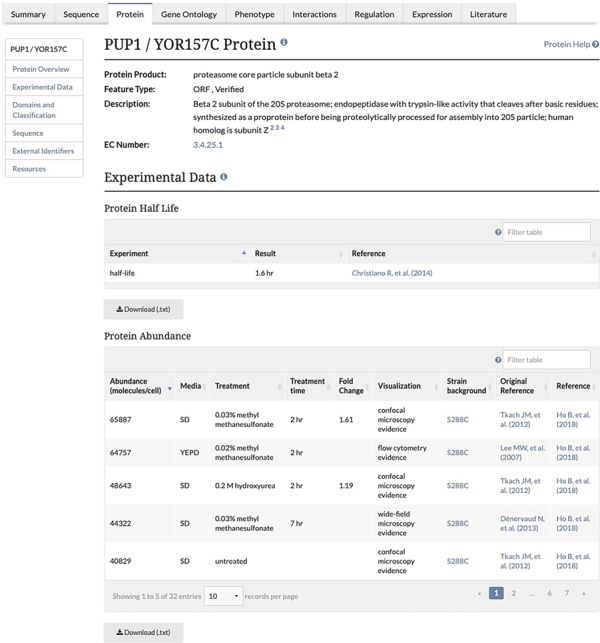
Experimental Data Section of the Protein Page. This section of the Protein page contains two tables, one containing protein half-life data and the second containing the protein abundance data, and associated metadata, along with the original reference and the reference for the combined unified dataset. This table is both sortable and filterable.

## Data, metadata and ontologies

We focused our curation efforts on the unified dataset obtained from the Brown lab (26). This paper contains abundance data collected from the unified dataset published by the Brown lab in Ho *et al.* (26), where protein abundance data from 21 separate previously published proteomic studies were collected and analyzed. These previous studies had generated protein abundance values by any one of several independent methods, including mass spectrometry, GFP tagging coupled with either fluorescence microscopy or flow cytometry and tandem affinity purification coupled with immunoblot analysis. Since the unit space of the original data was either relative (abundance units) or absolute (molecules per cell), Ho *et al.* (26) used mode-shift normalization and scaling to convert all measurements of protein abundance from these publications into the intuitive units of molecules per cell. After filtering values to remove background autofluorescence from fluorescence microscopy-based studies, they removed low-abundance GFP-fusion protein signals, reducing coverage of the unified dataset from 97% to 92% of the proteome, which represented 5391 proteins and improved correlation with the calibration dataset (26). In addition to the baseline data obtained under standard growth conditions, a subset of GFP-based studies containing abundance data gathered from cells exposed to various environmental stressors were also analyzed (16–20, 23–24). For treated cells, abundance values were also normalized and unified. When the value in stressed cells was more than two standard deviations away from the untreated average abundance, a fold change was also calculated (26).

**Figure 2:**
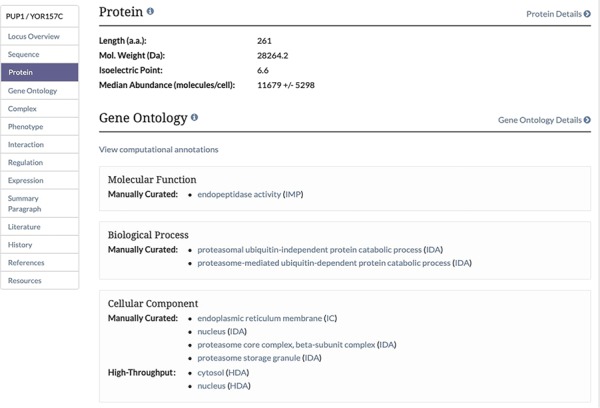
Protein Section of the Locus Summary Page. The protein section of the Locus Summary pages, located between the Sequence and Gene Ontology sections, contains the calculated median and MAD for the protein of interest expressed in molecules/cell in addition to basic sequence-derived information (length, molecular weight and isoelectric point). Median was calculated based on all values for a given protein from untreated cells, and MAD was calculated using the same values. When the median value was generated based on a single value, a MAD could not be calculated.

Metadata associated with the primary datasets used in Ho *et al.* (26) was reviewed and verified. This included the growth media, strain background, visualization method and, for treated cells, the treatment (including the concentration of chemical applied to the cells, when applicable), units and treatment time (26). To standardize the metadata representation and enhance computational analysis, several different ontologies were investigated. We used the Experimental Factor Ontology (EFO; https://www.ebi.ac.uk/efo/), originally developed to describe experimental variables for expression studies, to represent yeast growth media (28). We used the Evidence & Conclusion Ontology (ECO; http://www.evidenceontology.org), a controlled vocabulary that describes scientific evidence, to describe the various visualization methods (29). When chemical treatments were used to induce environmental stress, terms from the Chemical Entities of Biological Interest (ChEBI; https://www.ebi.ac.uk/chebi/), an ontology used to classify chemicals based on both shared structural features and activities, were used (30). Experimental treatments that involved nitrogen starvation or cellular quiescence were represented by Gene Ontology process terms (GO; http://www.geneontology.org) (31). Finally, strain backgrounds were recorded to document the genetic environment in which abundance was measured (https://wiki.yeastgenome.org/index.php/Commonly_used_strains).

### Integration of protein abundance data into SGD and YeastMine

To store this novel unified protein abundance data in the SGD database, we created a new database table containing fields for recording the protein entity to which the specific abundance value is associated, an identifier to indicate the annotation source, a taxonomy ID indicating the strain background and two reference IDs, one for the original data source and a second for the data normalization and unification paper. In addition, there are fields for the data value, data unit, assay ID (ECO identifier) and media ID (EFO identifier) for the various growth media used. For cells treated with an environmental stress-inducing agent or condition, the table contains fields for chemical ID (CHEBI identifier), concentration value, concentration unit, time value and time unit. For cases in which the environmental stress was not a chemical treatment, this was captured using a Gene Ontology process term and is stored as a GO identifier. The fold change is also included in cases where the value in stressed cells is more than two standard deviations from the untreated average abundance. Finally, a median value was calculated from all values for a given protein from untreated cells and was used to calculate MAD using all values from untreated datasets and a constant of *C* = 1 (26). In cases where the median value was generated based on data from a single study, the MAD could not be calculated. Scaled protein abundance data for untreated and treated cells were loaded into the SGD database, which houses the data, metadata, original and unified data reference, median and newly calculated MAD. Abundance data, metadata, median abundance and MAD values were also added to our YeastMine data warehouse, using the data integrated into the SGD database as the source.

## Accessing protein abundance data at SGD

Unified protein abundance data stored in our database are displayed on our public website on the Protein page in the experimental data section for each visualized protein-coding gene (Figure 1). The table is located below a table containing experimentally determined proteome-wide protein half-life data. This table, consistent with others on the SGD website, can be both filtered and sorted. The data in each table can be retrieved using the ‘Download’ button located under the table (Figure 1). The median and MAD values are displayed in the Protein section of the Locus Summary page, below sequence-derived values of protein length, molecular weight and isoelectric point (Figure 2).

Additionally, these data can be explored and downloaded from YeastMine (https://yeastmine.yeastgenome.org). Specifically, there are two templated pre-generated queries in the protein category; ‘Gene to Protein Abundance’, where abundance values for one or more proteins or a user customized list of proteins can be retrieved, and ‘Gene to Median Protein Abundance’, where median and MAD values for one or more proteins can be downloaded. These data are downloadable as tab- (.tsv) or comma-delimited text files (.csv), XML or JSON formats. Data is also downloadable using the YeastMine API (https://yeastmine.yeastgenome.org/yeastmine/api.do) or using SGD’s web services (e.g. https://www.yeastgenome.org/webservice/locus/S000000364/protein_abundance_details).

## Future directions

We are currently investigating ways to better visualize the protein abundance data. To provide users with an overview of the abundance values and variance of protein(s) of interest, we are exploring the use of scatter plots to visualize the abundance value or median value for a given protein relative to all other proteins and to visualize the effect of treatment with stress on relative abundance. We will also need to explore how best to update these data if additional abundance datasets become available.

## Notes

Website URL: https://www.yeastgenome.org
